# Does Effective Population Size Govern Evolutionary Differences in Telomere Length?

**DOI:** 10.1093/gbe/evae111

**Published:** 2024-05-21

**Authors:** Lyda M Brown, Mia C Elbon, Ajay Bharadwaj, Gargi Damle, Joseph Lachance

**Affiliations:** School of Biological Sciences, Georgia Institute of Technology, Atlanta, GA, USA; School of Biological Sciences, Georgia Institute of Technology, Atlanta, GA, USA; School of Biological Sciences, Georgia Institute of Technology, Atlanta, GA, USA; School of Biological Sciences, Georgia Institute of Technology, Atlanta, GA, USA; School of Biological Sciences, Georgia Institute of Technology, Atlanta, GA, USA

**Keywords:** comparative genomics, effective population size, genetic drift, natural selection, senescence, telomere length

## Abstract

Lengths of telomeres vary by an order of magnitude across mammalian species. Similarly, age- and sex-standardized telomere lengths differ by up to 1 kb (14%) across human populations. How to explain these differences? Telomeres play a central role in senescence and aging, and genes that affect telomere length are likely under weak selection (i.e. telomere length is a trait that is subject to nearly neutral evolution). Importantly, natural selection is more effective in large populations than in small populations. Here, we propose that observed differences in telomere length across species and populations are largely due to differences in effective population sizes. In this perspective, we present preliminary evolutionary genetic evidence supporting this hypothesis and highlight the need for more data.

**Significance**

Telomeres are protective caps at the ends of chromosomes that shorten with age, leading to cellular senescence. Despite this, telomere length varies substantially across taxa, and existing hypotheses are unable to fully explain this variation. In this perspective, we hypothesize that telomere-shortening alleles (and their deleterious effects) are more likely to accumulate in populations with fewer individuals.

## Natural Variation of a Health-Related Trait

Telomeres are the caps at the ends of chromosomes that function to protect ends from deterioration and provide chromosome stability (Shay 2018). They are composed of an array of repetitive DNA sequences (in vertebrates, TTAGGG) that shortens with every cell division, thus acting as a biological clock. It is largely accepted that telomere length (TL) declines in somatic cells with age, contributing to replicative senescence and cell death. Telomerase, a ribonucleoprotein that adds telomeric repeats to the 3′ end of telomeres, is generally expressed throughout life in small mammals but restricted after embryonic development in larger mammals, perhaps as a cancer-suppressing mechanism.

Shorter TL is generally associated with negative health outcomes, and selection pressures against critically short telomeres are likely to be stronger than selection pressures against extremely long telomeres. However, both extremes have been associated with increased risks of cancer (Julin et al. 2015; Jafri et al. 2016; Gong et al. 2020), leading some researchers to suggest that TL may be under stabilizing selection in long-lived species (Risques and Promislow 2018). That said, this *Perspective* largely focuses on the negative impact of short telomeres.

Lengths of telomeres vary across a range of biological scales. TL and age-dependent rates of telomere decay differ substantially between species (Remot et al. 2022). Among mammals that belong to the same order, TL can vary by as much as an order of magnitude (Gomes et al. 2011). The maximum number of times a cell can divide before becoming senescent also varies across mammalian species (Röhme 1981). Considerable variation in TL exists in humans both within and between populations. In humans, exposure to environmental contaminants and pollutants, physical activity, and quality of diet have been found to affect TL (López-Otín et al. 2013; Arsenis et al. 2017; Canudas et al. 2020). Many TL-associated variants exist, and their allele frequencies differ across human populations. This contributes to variation in TL. On average, African Americans have longer TL than Europeans, but shorter TL than sub-Saharan Africans (Hansen et al. 2016). It has been posited that human TL is a target of selection and that longer TL was ancestral in humans (Hunt et al. 2020).

Given the importance of telomeres with respect to cellular aging and disease, what can explain these differences in TL? We address this question by describing multiple life history traits that are associated with TL, before introducing a novel evolutionary hypothesis that invokes the relative efficacy of natural selection in large and small populations. We then highlight preliminary evidence in support of our hypothesis while addressing the need for more data.

## Associations with Life History Traits

Trade-offs may exist between TL and other life history traits within the same species. Studying Atlantic silversides (*Menidia menidia*), Gao and Munch (2015) found evidence that high fecundity was positively correlated with both reduced life span and shorter TL. Similarly, Walter et al. (2007) demonstrated an association between long telomeres and reduced fertility in *Drosophila melanogaster* females. In humans, the rate at which TL shortens is significantly faster in early life than in adulthood (Kuo et al. 2019), and TL may depend on paternal age at conception (Broer et al. 2013).

Comparative approaches reveal further correlations between TL and life history traits. Smaller mammals tend to have longer telomeres (Gomes et al. 2011). Related to this idea, Gomes et al. (2011) argued that trade-offs in the energetic costs of maintaining telomeres could account for substantial variation in TL among mammalian species. It has also been suggested that TL can shorten due to increased energy demands in times of stress (Casagrande and Hau 2019). However, it has been argued that cellular energetic costs are likely to yield minimal fitness effects in large multicellular eukaryotes (Lynch and Marinov 2015). Body temperature and the evolution of homeothermy may also contribute to differences in TL across taxa (Gomes et al. 2011). We also note that the prevalence of malignant neoplasms is higher in mammalian species that have longer telomeres (Pepke and Eisenberg 2021). In addition to TL, age-related rates of telomere shortening also vary across taxa, and this genetic trait has been shown to predict species-level differences in life span better than initial TL, body weight, and heart rate (Whittemore et al. 2019).

Although multiple life history traits are correlated with TL, many of the above relationships between TL and life history traits are taxon specific (Olsson et al. 2018). Existing explanations for TL variation are unsatisfactory as they lack a broad evolutionary perspective. Importantly, genes that are associated with TL exhibit signatures of weak selection over phylogenetic timescales (Pontremoli et al. 2018; Saint-Leandre and Levine 2020), and TL appears to have experienced polygenetic selection in human populations (Hansen et al. 2016).

## An Evolutionary Genetic Explanation for TL Variation

### Our Central Hypothesis

We propose that variation in TL across species and populations is largely governed by differences in effective population size.

### The Importance of *N_e_*

**Fig. 1.**
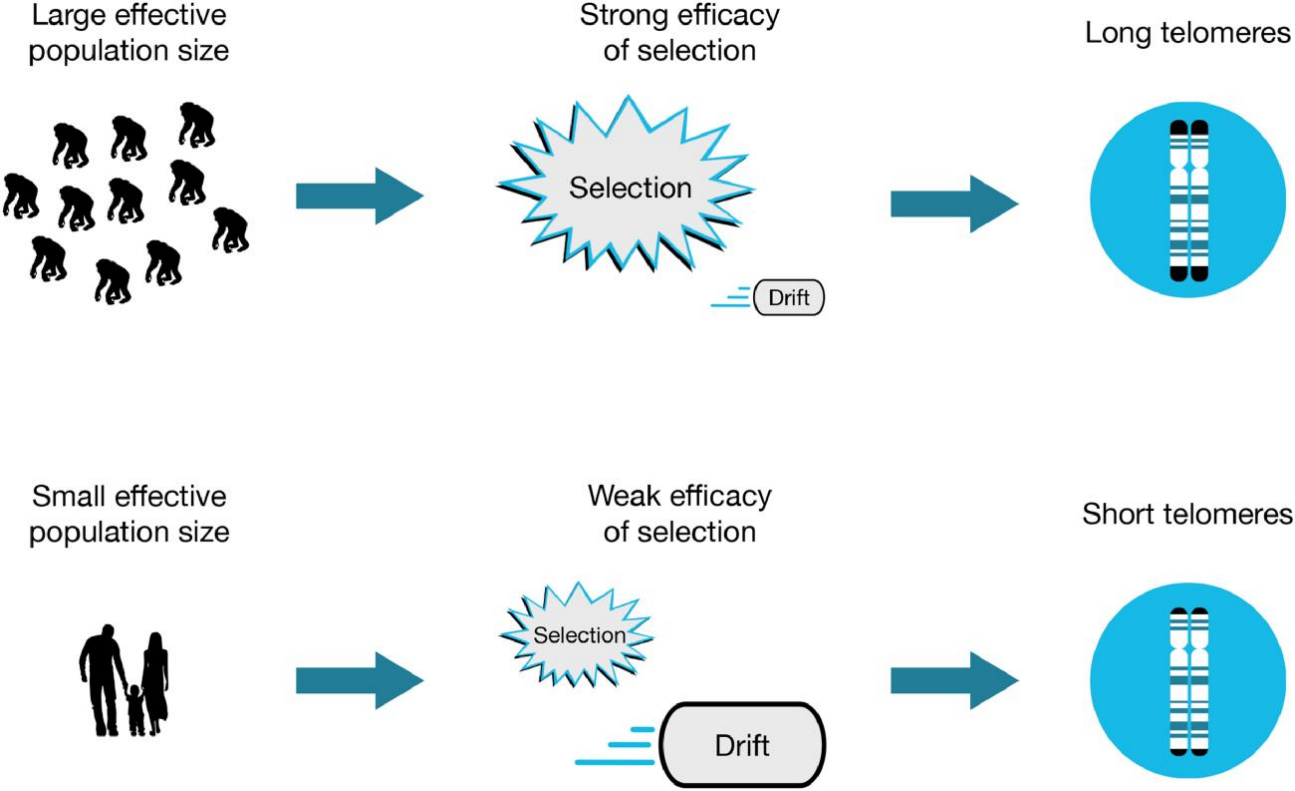
Schematic showing how effective population size can impact TL. Natural selection drives evolution when population sizes are large, which can result in the maintenance of long telomeres. Genetic drift plays a more important role when population sizes are small, which can result in the accumulation of slightly deleterious alleles that are associated with short telomeres.

Like other aspects of genomic architecture (Lynch 2007), TL is subject to evolutionary processes like natural selection and genetic drift (changes in the frequencies of different alleles in finite populations due to random chance). Critically, the relative importance of natural selection and genetic drift depends on effective population size (*N_e_*), the idealized population size that behaves the same way with respect to drift as a population of size *N*. (Charlesworth 2009). When few individuals contribute to a gene pool, random chance plays a large role in determining whether alleles increase or decrease in frequency. By contrast, when population sizes are large, the evolutionary fates of individual alleles are largely driven by natural selection (Fig. 1). Per Tomoko Ohta's nearly neutral theory of evolution, slightly deleterious mutations can reach fixation when population sizes are small (Ohta 1973). Because the efficacy of selection depends on the product of *N_e_* and the selection coefficient (Ohta 1992; Hughes 2008), alleles that are associated with short telomeres (and their weakly deleterious effects) are more likely to accumulate in populations with fewer individuals.

Do recent or historic population sizes matter more for the evolution of TL? It takes multiple generations for deleterious telomere-shortening mutations to accumulate after a population bottleneck. Similarly, species that have experienced recent explosive growth may still harbor short telomeres, as efficient purging of deleterious TL-associated alleles in large populations is not an instantaneous process. Because of this, we expect TL variation to be shaped more by long-term historic population sizes rather than recent population sizes. We note that *N_e_* can be calculated from polymorphism data (Charlesworth 2009; Wang et al. 2016), as smaller populations contain less genetic diversity, and that historical population sizes can be inferred via sequentially Markovian coalescent approaches (Mather et al. 2020).

## Plausibility of Our Hypothesis

### Preliminary Evidence from Nonmammalian Species

TL varies a considerable amount in nonmammalian vertebrate species. Short telomeres are observed in sharks and rays (∼3 kb) and in swordtail fish species (2 to 6 kb) (Rocco et al. 2001; Downs et al. 2012). By contrast, adult zebrafish have relatively high TL, ranging from 16 to 25 kb (Anchelin et al. 2011). TL variation has also been studied in lizards (Olsson et al. 2010; Fitzpatrick et al. 2021). Sampling 10 natural populations of *Zootoca vivipara*, Dupoué et al. (2017) found that lizard populations with a higher risk of extinction had smaller telomeres than populations that were nonthreatened.

Among bird species, the relationship between TL and *N_e_* is less clear. Here, we focus on three related species pairs that have *N_e_* estimates from 100,000 yr ago (Germain et al. 2023) and known TL (Tricola et al. 2018). Consistent with our hypothesis, barn swallows (*Hirundo rustica*, *N_e_* = 3.6 × 10^5^, TL = 8.7 kb) have both a smaller historical *N_e_* and shorter telomeres than white-rumped munia (*Lonchura striata*, *N_e_* = 5.7 × 10^5^, TL = 16.36 kb). Examining two species of seabirds, we note that Adélie penguins (*Pygoscelis adeliae*, *N_e_* = 3.7 × 10^4^, TL = 7.35 kb) and northern fulmars (*Fulmarus glacialis*, *N_e_* = 2.3 × 10^4^, TL = 14.25 kb) have similar *N_e_* but widely different lengths of telomeres. Lastly, thick-billed murre (*Uria lomvia*, *N_e_* = 14.4 × 10^4^, TL = 7.7 kb) and black guillemot (*Cepphus grylle*, *N_e_* = 5.6 × 10^4^, TL = 14.59 kb) show the opposite trend of what we would expect, as the species with a larger *N_e_* has shorter telomeres. Notably, many species of birds have ultralong telomeres ranging in size from a few hundred kilobases to 1 to 2 Mb (Delany et al. 2000; Atema et al. 2019). The function of these ultralong telomeres is not well characterized, but they have been shown to localize on gene-rich microchromosomes (Nanda et al. 2002).

### Preliminary Evidence from Mammals

**Fig. 2.**
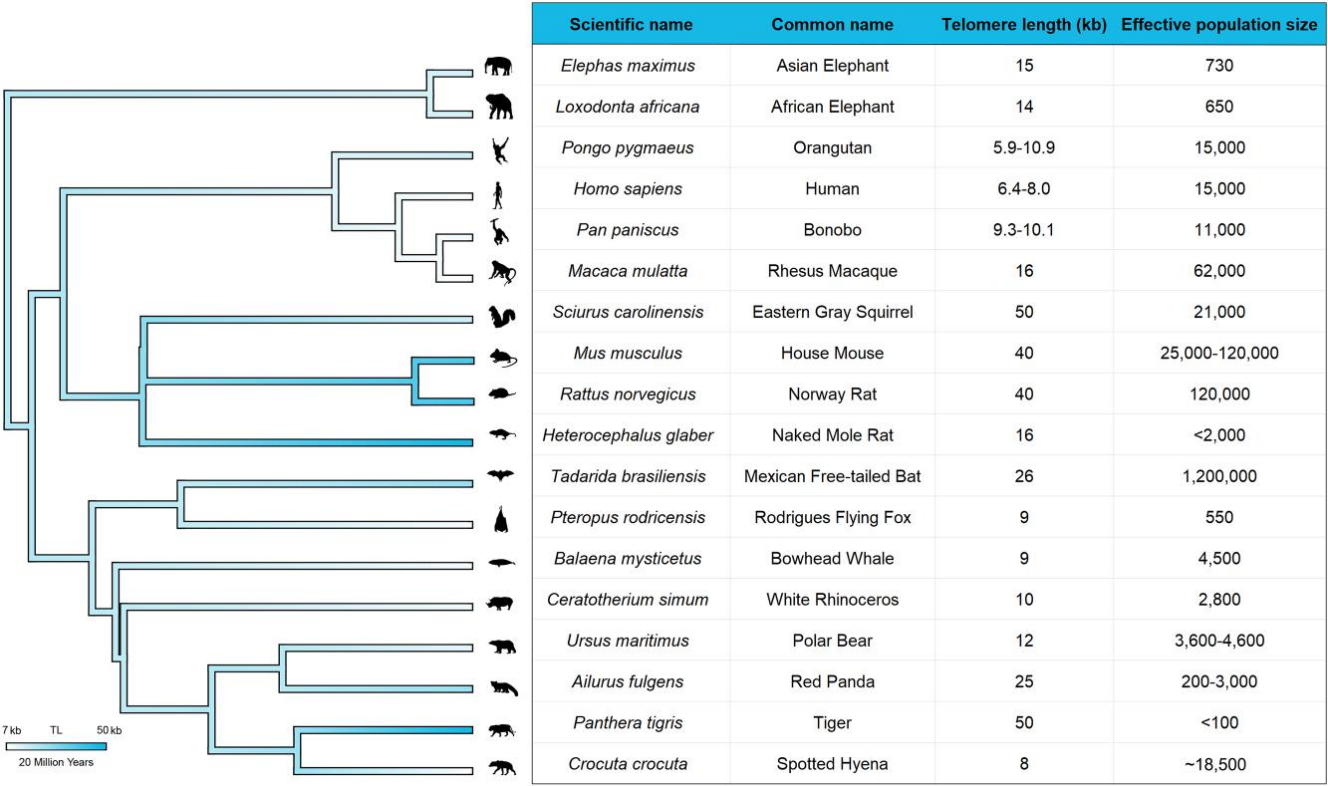
Adult TL and effective population sizes for 18 different species of mammals. Sources of TL data: (Gomes et al. 2011; Hansen et al. 2016). TimeTree (Kumar et al. 2017) and MEGA (Kumar et al. 2018) were used to obtain divergence times and generate the mammalian phylogeny. Figure 1 from Gomes et al. (2011) served as an inspiration for this figure. In most cases, TL estimates are from a single adult individual of indeterminant age, underscoring the need for additional data and further studies of TL variation across taxa. When needed, a mammalian mutation rate of 7.97 × 10^-9^ mutations per site per generation was used to infer *N_e_* from observed heterozygosity (Bergeron et al. 2023). Sources of *N_e_* data: (Reeve et al. 1990; O’Brien et al. 2007; Okello et al. 2008; Cronin et al. 2009; Russell et al. 2011; Phillips et al. 2012; Nater et al. 2013; Prado-Martinez et al. 2013; Singh et al. 2014; Deinum et al. 2015; Signorile et al. 2016; Sharma et al. 2018; Westbury et al. 2021; Ullrich and Tautz 2020).

Not only do mammalian species have substantial variation in TL, but their population sizes also vary by multiple orders of magnitude. In a landmark study, Gomes et al. (2011) showed that TL and other life history traits vary substantially across mammalian taxa. Building on this work, we combined estimates of adult TL for 18 mammalian species (Gomes et al. 2011; Hansen et al. 2016) with previously published estimates of *N_e_* (Fig. 2). Because population bottlenecks can yield evolutionary mismatches between a species’ ancestral *N_e_* and its current population size, we excluded *N_e_* estimates for domesticated species in our comparisons of mammalian data. As seen in Fig. 2, TL is an evolutionary labile trait—there are multiple branches where telomeres have either shortened or elongated. We also note that many large *N_e_* species also have long telomeres, a pattern that is consistent with there being more efficient selection against short telomeres in larger populations.

Comparisons between related species of mammals yield further insights. Despite both species belonging to the order Chiroptera, flying foxes (*Pteropus rodricensis*) and Mexican free-tailed bats (*Tadarida brasiliensis*) have a large difference in TL (9 vs. 26 kb, respectively), which may be due to large differences in *N_e_* (550 vs. 1,200,000). Among primates, rhesus macaques (*Macaca mulatta*) have much longer TL than great apes (Fig. 2). This pattern is consistent with the large *N_e_* of macaques. Similarly, humans are known to have shorter telomeres than chimpanzees (Kakuo et al. 1999) as well as smaller historic population sizes (Chen and Li 2001). When pairs of closely related species have similar *N_e_*, we would expect them to also have similar TL, a pattern that was observed for African and Asian elephants (*Loxodonta africana* and *Elephas maximus*). Similarly, both house mice (*Mus musculus*) and Norway rats (*Rattus norvegicus*) have large TL as well as large *N_e_* (Fig. 2). We note that some pairwise comparisons are not congruent with our hypothesis. Despite having a small *N_e_*, tigers (*Panthera tigris*) have much larger telomeres than spotted hyenas (*Crocuta crocuta*). This unexpected pattern may be related to greater rates of telomere attrition with age in felines compared with other mammals (Brummendorf et al. 2002).

One counterintuitive trend is that short-lived mammalian species tend to have longer telomeres than long-lived species (Risques and Promislow 2018). This paradoxical pattern is the opposite of what might be expected given the important role of telomeres with respect to aging and senescence. However, we note that long-lived species also tend to have smaller population sizes. Our evolutionary hypothesis that telomere-shortening alleles (and their deleterious effects) are more likely to accumulate in populations with fewer individuals may help resolve this biological paradox.

### Preliminary Evidence from Human Populations

The same evolutionary processes that shape the genomes of other mammals also apply to our own species and measurements of TL across human populations. In 2008, a genetic study of 2,454 individuals found that African Americans have longer telomeres on average than European Americans (Hunt et al. 2008). More recently, Hansen et al. (2016) measured leukocyte TL (LTL) by Southern blot analysis in 90 Europeans, 97 African Americans, and 100 East Africans. After correcting for age and sex of study participants, they found that Europeans had a mean LTL of 7.21 kb, African Americans had a mean LTL of 7.42 kb, and East Africans had a mean LTL at 7.82 kb. Notably, populations with larger telomeres also tend to have larger effective population sizes. Building on this prior work, Hunt et al. (2020) conducted a comprehensive study of LTL in 1,295 sub-Saharan Africans. Among sampled African populations, Khoisan hunter-gatherers from Botswana had the longest LTL (Hunt et al. 2020). Intriguingly, the Khoisan are descendants of an ancient population in Southern Africa that historically had one of the largest *N_e_*, consistent with our hypothesis (Kim et al. 2014).

Genome-wide association studies have identified many loci that affect TL, telomere maintenance, and aging in humans (Buniello et al. 2019; Dorajoo et al. 2019). In humans, heritability estimates of LTL range from 44% to 86% (Njajou et al. 2007; Broer et al. 2013). Recently, two large-scale studies have yielded polygenic risk scores (PRS) for TL (Li et al. 2020; Taub et al. 2022). In a meta-analysis of over 78,000 European individuals, Li et al. (2020) identified 20 genetic variants that are associated with TL. Similarly, analyzing data from over 109,000 TOPMed participants, Taub et al. (2022) identified 59 genetic variants that are associated with TL. These two studies reveal that TL is a polygenic trait, which suggests that any selection acting on individual loci is likely to be weak. Many of the top variants that influence TL in humans colocalize with telomerase genes like *TERT*, *TERC*, and *NAF1*.

Intriguingly, Taub et al. also reported ancestry differences in TL. Applying their PRS to individuals in the BioVU biobank at Vanderbilt University, they found that African Americans have larger predicted TL than European Americans, *P* < 0.05, Welch's two-sample *t*-test (Taub et al. 2022). These findings align with our hypothesis that populations with larger *N_e_* are more likely to have longer telomeres.

## Testable Predictions and Future Directions

Our evolutionary hypothesis yields testable predictions. For example, because Sumatran orangutans (*Pongo abelii*) have a larger *N_e_* than Bornean orangutans (*Pongo pygmaeus*) (Prado-Martinez et al. 2013), we speculate that they have longer telomeres. Similarly, we would expect black bears (*Ursus americanus*) to have longer telomeres than polar bears (*Ursus maritimus*) due to their larger *N_e_* (Cahill et al. 2013). Another future research avenue involves examining TL in species that have experienced recent changes in population size. Because it takes many generations for new deleterious mutations to accumulate in bottlenecked populations, we hypothesize that domesticated animals and their wild counterparts are likely to have similar telomere sizes.

Going forward, a major challenge is the need for more data. Most of what is known about telomere genetics in humans comes from European populations. Because of this ascertainment bias, loci that affect TL in other populations remain undiscovered. There is a clear need to conduct genome-wide association studies of TL in diverse (non-European) human populations. Similarly, despite recent advances in comparative genomics like the Zoonomia Project (Zoonomia Consortium 2020), accurate TL estimates are only available for a limited number of species. Even when TL estimates are available, the ages of samples are not always known. In addition, the use of different approaches to measure TL (see Box 1) can make comparisons between taxa challenging. Because of this, there is a need to quantify the relative lengths of telomeres in different species using shared methodologies. An additional need involves quantifying genetic diversity in wild populations, as inbreeding is likely to skew estimates of *N_e_* obtained from zoo animals (Dyson et al. 2022). Future studies of TL diversity across the tree of life will also benefit from reporting life history characteristics of each individual, e.g. age, sex, and body weight. Other exciting avenues of research include comparative transcriptomics and studying telomerase activity across a spectrum of ages (Dugdale and Richardson 2018).

Box 1. **Current methods for measuring TL**

TL can be measured directly by digesting genomic DNA with a cocktail of restriction enzymes that leave the repetitive TTAGGG sequence intact as terminal restriction fragment lengths (TRFs). Notably, TRFs contain both the telomere caps and adjacent subtelomeric region, leading to an overestimate of TL. Although TRF detection by Southern blot was developed over 30 years ago, it remains the gold standard for measuring TL. Two drawbacks of this method are the large amount of starting material required (3-μg high-quality gDNA) and lack of sensitivity to detect the shortest telomeres (Harley et al. 1990; Kimura et al. 2010).

Single telomere length analysis (STELA) targets telomeres from a specific chromosome with PCR followed by Southern blot analysis. A limitation of the application of STELA is that not all chromosomes have unique sequences for designing PCR primers (Baird et al. 2003). Telomere shortest length assay is another PCR/Southern blot technique that measures the shortest telomeres from all chromosomes ends within a limited range (1 to 18 kb) (Lai et al. 2017).

High-throughput methods include quantitative PCR, whereby the number of telomere copies relative to a single copy gene provides an indirect measurement of TL (Cawthon 2002). Zooming in, individual telomeres of cells arrested at metaphase can be quantified under a microscope via quantitative fluorescence in situ hybridization (Q-FISH) that uses a peptide nucleic acid probe complementary to TTAGGG (Lansdorp et al. 1996). An adaptation of this method that adds flow cytometry (flow FISH) allows select populations of cells to be measured (Rufer et al. 1998). The single telomere absolute-length rapid assay uses a fluidic chip to measure individual telomeres with digital real-time PCR and includes a synthetic telomere standard for a direct measurement of length. It requires less than 1 ng of starting material, 3-h processing time, and an impressive dynamic range of 0.2 to 320 kb. Software has also been developed to estimate the average TL from whole-genome sequencing data with assembled genomes (Ding et al. 2014).

Here, we focused on how TL varies between populations and species with different *N_e_*. This perspective helps explain the paradoxical pattern of shorter TL in long-lived species. Despite the utility of our novel evolutionary hypothesis, we note that many different factors contribute to variation in TL; *N_e_* does not explain everything. It is also important to keep in mind that other aspects of telomere biology can vary across species, including telomerase activity and age-related rates of telomere decay. Although molecular approaches (e.g. examining difference in telomerase expression) can yield mechanistic insights, adopting an evolutionary genetic perspective can help explain why TL varies in the first place.

## Data Availability

No new data were generated or analyzed in this study.
